# Effects of tDCS and tACS on operant tactile training: investigating individual differences in neuromodulation efficacy

**DOI:** 10.1007/s00221-026-07346-2

**Published:** 2026-07-06

**Authors:** Emily Gebert, Tran Ngoc-Huong Quan, Ahmed A. Karim, Ben Godde

**Affiliations:** 1 School of Business, Social & Decision Sciences, Constructor University, Campus Ring 1, 28759 Bremen, Germany; 2 Department of Psychiatry and Psychotherapy, University of Tübingen, Tübingen, Germany

**Keywords:** Tactile perceptual learning, Non-invasive brain stimulation, Transcranial direct current stimulation, Transcranial alternating current stimulation, Electroencephalography

## Abstract

Tactile perceptual learning—the improvement of tactile discrimination abilities through experience or training—is fundamental to sensory rehabilitation and skill acquisition. Non-invasive brain stimulation techniques have emerged as promising tools to modulate somatosensory cortical excitability and plasticity, thereby enhancing tactile perceptual learning. Here we present two experiments in which we further examined the effectiveness of transcranial direct and alternating current stimulation on tactile operant learning in a grating orientation discrimination task (GOT). While in experiment 1 we compared the online and offline training effects of tDCS to sham stimulation and tDCS effects without training, in experiment 2, we contrasted the effects of tDCS and tACS on tactile training and examined whether training effects could be predicted by baseline oscillatory brain activity as measured with electroencephalography. In both experiments, participants improved their discrimination performance during training independent of group (experiment 1) or condition (experiment 2), even when controlling for pre-test thresholds. Offline effects of training were inconsistent between experiments. Irrespective of stimulation conditions (tDCS, tACS, sham), training effects could be predicted by baseline brain oscillatory patterns. Higher frontal theta-beta power ratio predicted stronger online training effects, particularly in the tDCS condition. Offline effects of tDCS and tACS combined with tactile training could be predicted by centro-parietal alpha power at pre-test. Interestingly, in experiment 2, participants revealed lower GOT thresholds during training in the tDCS than in the tACS condition. One might speculate that tACS interferes with task-related alpha desynchronization during training. This option requires further testing in future experiments.

## Introduction

Tactile perceptual learning—the improvement of tactile discrimination abilities through experience or training—is fundamental to sensory rehabilitation and skill acquisition. Non-invasive brain stimulation (NIBS) techniques, such as repetitive transcranial magnetic stimulation (rTMS), transcranial direct current stimulation (tDCS), and transcranial alternating current stimulation (tACS), have emerged as promising tools to modulate cortical excitability in the somatosensory cortex (SI) and the combination of NIBS techniques with behavioral interventions has revealed potential in promoting neuroplasticity and learning (Cramer et al. 2011).

Facilitating effects of (r)TMS on tactile discrimination performance and tactile perceptual training have been well established (Ragert et al. 2003; Tegenthoff et al. 2005). Moreover, we previously demonstrated that high frequency rTMS over SI combined with tactile training facilitates spatial discrimination learning (Karim et al. 2006). Notably, transcranial electrical stimulation has also gained attention as an accessible, comfortable method to modulate brain activity across cognitive, motor, and perceptual domains. For example, anodal tDCS applied over the primary somatosensory cortex (S1) has been shown to enhance tactile spatial discrimination in healthy individuals and stroke patients (Ragert et al. 2008; Fujimoto et al. 2014, 2016). When paired with operant training, tDCS may facilitate both short- and long-term learning (Hilgenstock et al. 2016).

In a meta-analysis, we evaluated the results of nine randomized controlled trials with 159 participants, which investigated the effects of a tDCS intervention on tactile discrimination in a pre/post treatment design comparing experimental to control (sham stimulation) groups (Khalil et al. 2023). In eight of these nine studies, tactile spatial discrimination was measured with the grating orientation discrimination task (GOT), a standard measure of tactile spatial acuity (Van Boven and Johnson 1994). Overall, an improvement in tactile perception, characterized by a decrease in discrimination thresholds, after tDCS in the experimental as compared to the control group, was observed in six of the nine studies (Fujimoto et al. 2014; Godde et al. 2020; Saito et al. 2019; Swissa et al. 2022; Ragert et al. 2008; Yau et al. 2014). However, while most reports claimed positive effects of tDCS, i.e., a decrease in detection or discrimination thresholds, three studies reported that tDCS did not affect tactile discrimination perception compared to the control group (Fujimoto et al. 2017; Hilgenstock et al. 2016; Hirtz et al. 2018). Overall our meta-analysis revealed a small but significant effect, however, a high degree of heterogeneity was reported (Khalil et al. 2023). A recent review confirmed such divergent results for several different tactile tests, for example, detection and spatial, temporal, or amplitude discrimination tasks (Sasaki et al. 2025). Besides differences in experimental protocols, stimulation parameters or target regions, individual variations might also contribute to the variability observed in studies utilizing tDCS for enhancing tactile perception (Polanía et al. 2018). Also, online effects during stimulation must be distinguished from offline effects maintained after the end of the stimulation period (Hilgenstock et al. 2016).

It seems plausible that reported inconsistencies in the effects of transcranial electrical stimulation on tactile performance and learning might be related to individual differences in baseline brain activity and learning thresholds. Resting state EEG is a useful measurement to characterize brain states and to relate them to behavioral variance in both clinical patients and healthy individuals (Coombes et al. 2005; Pitto et al. 2011). However, only few studies investigated the predictive role of resting state EEG on perceptual learning. In a visual perceptual learning experiment, resting-state alpha was larger for good learners both before and during learning (Muller-Gass et al. 2017). Freyer et al. (2013) reported that up to 64% of the observed learning variability in a tactile perceptual learning tasks could be predicted by pre-learning parietal alpha power and stimulus-induced central alpha desynchronization during learning. Beta oscillations neither before nor during training did predict learning outcome. Brickwedde et al. (2019) confirmed that high pre-learning alpha levels are a requirement for gating tactile perceptual learning. Mostly outside the tactile domain also other brain oscillatory parameters have been researched. Resting-state beta oscillations (13–14.5 Hz) were shown to predict language proficiency and learning success in young (Kliesch et al. 2022) and older adults learning a second language (Kliesch et al. 2021). Another study found that lower levels of resting-state theta power (6–7 Hz) were associated with better implicit visuospatial-motor sequence learning (Lum et al. 2023). In cognitive training, a higher alpha/theta ratio was associated with better outcomes (Juras et al. 2025). Own data revealed that successful dynamic balance training could be predicted by higher theta and lower beta power in the resting state EEG (Imani and Godde 2025).

Current research has further highlighted the potential of tACS to enhance tactile perception through frequency-specific entrainment (Herrmann et al. 2013). Unlike tDCS, tACS uses a sinusoidal alternating current that does not result in net polarization of neurons (Gholamali Nezhad et al. 2024). Instead, its effects are entirely dependent on the frequency of the applied current. Remarkably, applying tACS over SI can induce phantom tactile sensations in the contralateral hand in a highly frequency-dependent manner (Feurra et al. 2011). Their research has identified specific frequency bands that are most effective for inducing these sensations: the alpha band (10–14 Hz) and the high gamma band (52–70 Hz), with a weaker effect also observed in the lower beta band (16–20 Hz) (Feurra et al. 2011). The alignment of these effective frequencies with rhythms involved in sensorimotor activity provides compelling evidence that tACS directly interacts with the brain’s natural oscillatory dynamics (Antal and Paulus 2013; Helfrich et al. 2014; Orendáčová and Kvašňák 2021). Alpha-frequency tACS has been shown to improve cognitive and perceptual performance by synchronizing intrinsic alpha rhythms (Millard et al. 2024). Alpha and high gamma entrainment via tACS improved tactile perception in the contralateral hand (Feurra et al. 2011). Saito and colleagues (2021) confirmed alpha-frequency tACS effects on improving tactile performance, with greater effects seen in those having lower baseline alpha power. However, no effects were found among older individuals with low alpha power (Fresnoza et al. 2018). On the contrary, in a preliminary study Sliva et al. (2018) observed worse tactile detection after alpha-frequency tACS compared to sham stimulation, suggesting temporary perceptual suppression.

Contributing to this ongoing discussion, we present two experiments in which we further examined the effectiveness of tDCS and tACS in operant tactile learning. Our experiments served three major aims:

First, in experiment 1 we evaluated whether tDCS would be as effective as rTMS in facilitating operant learning and thus could be a cheaper, easier to apply, and more mobile alternative to TMS in research and clinical settings. To this aim we replicated as closely as possible with tDCS our previous TMS study (Karim et al. 2006) in which we demonstrated that concurrent trains of 15 Hz rTMS facilitated online and offline training effects in a tactile grating orientation discrimination task (GOT). Similar to the TMS study, participants received 20 min of GOT training with concurrent tDCS over the contralateral primary somatosensory cortex (SI) and changes in GOT performance were assessed online (during training) and offline (after training).

Second, building on the fast-emerging literature on tACS, we tested the assumption that tACS would be as effective or even more effective as tDCS. To this aim, in experiment 2, we compared the effects of alpha-tACS and anodal tDCS over the contralateral SI on GOT performance and operant training applied to the left ring finger. While effects for tDCS seem to be well established (Khalil et al. 2023; Sasaki et al. 2025), alpha-tACS over the somatosensory cortex has been shown to effectively entrain alpha-frequency oscillations in the underlying cortical region (Gundlach et al. 2017) and to improve tactile performance (Saito et al. 2021). Direct comparisons of how tDCS and alpha-tACS affect tactile training are currently lacking. Given the inconsistent findings in the literature, this study takes an exploratory approach to compare the effectiveness of both methods.

Third, to further examine the predictive role of individual brain oscillation patterns for effects of tDCS and tACS on tactile learning, in experiment 2, we used EEG to measure resting state brain oscillations before training. We focused our analysis on centroparietal alpha power and frontal theta-beta power as specific predictors for tactile perceptual learning through sensory gating mechanisms and top-down inhibitory control of task-irrelevant brain resources, respectively (Freyer et al. 2013; Wei et al. 2024). We expected that higher alpha power and larger theta-beta ratios would predict learning outcomes in both tDCS and tACS conditions. Saito and colleagues (2021) reported improving tactile performance after alpha-frequency tACS, with greater effects seen in those having lower baseline alpha power. Therefore we further assumed that the effect of tACS could be moderated by the individual peak alpha frequency (PAF) and its difference to the tACS frequency.

## Methods and procedures

### Participants and sampling

Potential participants were approached through convenience sampling from the student population on the Constructor University Bremen campus. No monetary compensation or other incentives for study participation were given. The study was approved by the Constructor University Ethics Committee and conducted in accordance with the declaration of Helsinki. All participants were screened for neurological conditions and provided informed consent before the experiment. The exclusion criteria included neurological disorders, drug use, abnormal brain conditions, and left-handedness, as determined by the Edinburgh Handedness Inventory Questionnaire (Oldfield 1971). After initial screening, *N* = 36 participants (25 female, 11 male), aged 18 to 24 years participated in *experiment 1* and *N* = 17 participants were invited to *experiment 2*. Of those 17, three participants were excluded during experiment 2 due to incompatibility or irritation with the stimulation device. After exclusions, data of *N* = 14 participants (8 female, 6 male), aged 18 to 23 years, were included in the data analysis.

Prior to their appointment, each participant was sent a digital copy of the study information. On the day of the experiment, the participant filled in and signed a physical copy of the screening form. Once the eligibility was confirmed, written informed consent was given. Prior to starting the experiment, the respective study design for each group was explained verbally and the participant was given the opportunity to ask final questions or raise concerns.

## General procedures and materials

For *experiment 1*, participants were randomly allocated to three different experimental groups via a random number generator program written in python. Each group consisted of 12 participants. Group 1 (experimental group) received tDCS stimulation and training, group 2 (sham control group) received training plus sham stimulation and group 3 (no training control group) only received the tDCS stimulation without training. Pre- and post-test designs remained equal for all three groups.

In *experiment 2*, each participant completed two sessions (tDCS and tACS) within a one-week period, following a crossover design. The one-week interval was implemented to avoid carry-over effects of the stimulation (Andrews et al. 2011). The order of the protocols was counterbalanced across participants. On the day of each session, participants completed a pre-test on the Grating Orientation Task (GOT), received either tDCS or tACS during GOT operant training, and then completed a post-test on GOT. Resting-state EEG was recorded at the beginning and end of each session.

## Pre- and post-tests

Each participant performed pre- and post-tests to assess their spatial discrimination performance at the left ring finger with a tactile grating orientation task (GOT). The GOT consisted of eight hemispherical plastic domes that had regular gratings cut into their surfaces, resulting in parallel bars and grooves of constant width. The width of the bars and grooves ranged from 3.0 mm to 0.25 mm. To control for equal pressure and timing of each trial, the touch was administered passively by the experimenter, while strapping the participant’s non-dominant left hand into a custom-built device that allowed the experimenter to move the participants’ hand up and down. The hand was strapped in a way so that the tip of the left ring finger touched the dome when pressing the device down. The experimenter oriented the domes with their gratings either horizontally or vertically in relation to the participant’s hand. Each touch lasting for about 1.5 s and indenting the skin by 1–2 mm. The task of the participant, who was blindfolded, was to detect the orientation. After each trial, the participant’s answer was recorded on a corresponding trial protocol sheet. There was no feedback from the experimenter during pre- and post-tests.

In *experiment 1*, the pre- and post-test consisted of 20 blocks of 8 trials each, meaning that each of the 8 domes was applied once per block and 20 times in total. Horizontal (H) and vertical (V) orientations were pseudorandomly presented with equal probability for each block and the whole test. The test started with the 3.0 mm spaced dome and continued in a descending order until reaching the last dome with 0.25 mm spaced gratings. Pre- and post-test thresholds were calculated with the following formula (cf. Van Boven and Johnson 1994):

$$ T_{{G75}} = G{\text{ below}} + \left( {\frac{{0.75 - P{\text{ below}}}}{{P{\text{ above}} - P{\text{ below}}}}} \right) \times \left( {G{\text{ above}} - G{\text{ below}}} \right) $$

where

T_G75_ = threshold where the subject would have scored exactly 75% correct,

P_above/below_ = Probability for correct responses at smallest/largest grating width with more/less than 75% correct responses,

G_above/below_ = smallest/largest grating width at which probability for correct response was higher/lower than 75%.

In *experiment 2*, pre- and post-tests were employed with a staircase method, in which a dome was applied three times per block with different orientations, starting from the largest dome (3.0 mm). If there were two or more correct guesses, the participant moved up to the next level (smaller domes). If there were two incorrect guesses, the participant moved down to the previous level. Each move up or down created a turning point. The test continued until the sixth turning point was reached. Then, the average of the six turning points was calculated to determine the tactile threshold. No feedback was offered during the testing period. This procedure allowed us to reduce the time needed for testing.

## Training

For both experiments, the training consisted of 40 blocks of 8 trials each. During training, verbal feedback was applied (correct/wrong) after each trial. Starting with one grating larger than the threshold determined in the pre-test, the training was designed in a staircase method. If the participant made more than 25% mistakes per block, i.e., more than 2, the grating width was increased by one step. If 2 or less mistakes were made, the grating width was decreased by one step. The 40 blocks took around 20 min. The training average was calculated by taking the grating width in mm of the pre-test and subtracting it from the grating width of each block that followed. Then, the average of those values was calculated as the average change in threshold during training.

## Stimulation with tDCS / tACS

The tDCS, tACS or sham stimulation was conducted with the NeuroConn DC-Stimulator PLUS (neuroCare Group GmbH, Ilmenau, Germany). The electrodes were put into sponge-like pouches that were soaked with saline solution for better conductance and then placed on the scalp where they were fixed with elastic straps. To locate the right somatosensory cortex (contralateral to the tested left ring finger), first Cz and then C4 was determined after the 10/20 system (Jasper 1958). A small anodal electrode (size 5 × 7 cm; surface area 35 cm^2^) was placed with its centre ~ 2 cm behind C4 (Karim et al. 2006). In experiment 1, a large functionally ineffective reference electrode (size: 10 × 10 cm; surface area 100 cm^2^) was placed on the participant’s forehead. In experiment 2, due to incompatibility with the EEG electrode setup, the reference electrode was positioned over the back of the head. According to Nitsche and colleagues current density (current strength/electrode size) determines the efficacy of tDCS and by using a small stimulation electrode and a large reference electrode as in our experiments modulation of cortical excitability can be focused to the target area (Nitsche and Paulus 2000; Nitsche et al. 2007).

After adjusting the parameters on the device, the participant was informed about potential side effects and given instructions on how to communicate discomfort or pain one last time, to ensure their safety. The stimulation was then initiated. In cases where the impedance was too high to initiate the stimulation, more saline solution was added to the sponges, and scalp, hair and electrodes were adjusted, until the impedance decreased enough to start the stimulation.

In *experiment 1*, anodal stimulation (groups 1 and 2) was delivered to the right somatosensory cortex with a current set to 1 mA, with a ramp on- and off time of 20 s, and total stimulation time was set to 20 min. For the sham stimulation (group 3), the device only ramped up to 1 mA for 20 s and then turned off, while the participant was made to believe that it was running for the full 20 min.

In *experiment 2*, anodal tDCS stimulation was delivered during training the same way as in experiment 1 for exactly 20 min. In the tACS session, an alpha oscillation of 10 Hz was applied at a constant current intensity of 0.7 mA (peak to peak). A sinusoidal waveform without any direct current offset was used for stimulation. tACS was applied for 20 min during training, with 10 s of ramping up and down (Saito et al. 2021).

In both experiments, participants were blinded to the stimulation condition. In Experiment 1, the ramp-on/ramp-off procedure ensured that participants in the sham condition believed stimulation was running for the full 20 min. In Experiment 2, blinding is more difficult to guarantee in a crossover design, as participants experienced both stimulation types and may have detected differences in sensation between tDCS and tACS. No formal blinding check was administered after each session, which is acknowledged as a limitation. Regarding experimenter blinding, the person administering GOT training was aware of the stimulation condition; however, feedback during training was fully standardized and thresholds were derived from objective performance data, which substantially limits the risk of experimenter-induced bias.

### EEG acquisition and analysis (experiment 2)

In *experiment 2*, eyes-closed resting state EEG data were acquired for 2 min at the beginning and end of each session. Data was collected using a 32-channel active electrode system (ActiveTwo, BioSemi, Amsterdam, Netherlands), and electrodes were distributed according to the international 10/20 system (Jasper 1958). Data was collected from nine electrodes, Cz, C3, C4, Pz, P3, P4, Fz, F3, and F4. The Common Mode Sense (CMS) active electrode and the Driven Right Leg (DRL) were default reference electrodes. Electrode impedances were kept below 5 kOhm for all recordings.

Only data from the first EEG recording are included for analysis in this study. The EEG data were preprocessed and analyzed using the EEGLAB Toolbox (Delorme and Makeig 2004) in Matlab (version R2024a) (MathWorks, Natick, MA, USA). Data was preprocessed using a streamlined procedure with steps including resampling to 256 Hz, re-referencing to the average of all electrodes, band-pass filtering with a lower edge at 1.5 Hz and a higher edge at 30 Hz, and artifact reduction using independent component analysis (ICA). Power analysis was then performed with the eegstats plugin (Delorme 2024) with frequency bands defined as theta (4–7 Hz), alpha (8–12 Hz), low-beta (13–20 Hz), and high-beta (21–30 Hz). Oscillatory power was expressed as power spectral density (PSD) after the formula PSD [dB] = 10·log_10_(µV^2^/Hz). For further analysis centroparietal alpha power (cpAlpha) was calculated as the average of electrodes C3, Cz, C4, P3, Pz, and P4. Theta power and both, low -and high-beta power were strongly correlated and thus, frontal theta-beta ratio (fTBR) was calculated as the mean difference between theta and low-beta power at electrodes F3, Fz, and F4. Peak alpha frequency (PAF) was calculated as the frequency bin with the highest alpha power.

## Data management and analysis

Data was stored and managed in accordance with BIDS - Brain Imaging Data Structure Specification (Gorgolewski et al. 2016). Each participant was given a numerical code to anonymize the data. Data from the GOT was digitized and stored in the participant’s corresponding folder together with the EEG data (experiment 2) and demographic information. For *experiment 1*, one participant of the sham tDCS group was excluded from all analyses because the GOT threshold of this participant increased by a factor of 2 from the pre to the post test.

Statistical analyses were conducted with R in Jamovi version 2.6 (The Jamovi Project, 2025; R Core Team 2021). Longitudinal analysis of covariance using general linear modelling was conducted to test for online and offline effects of tDCS and tACS (Twisk et al. 2018). Effects were deemed significant with *p* < .05 for all statistical tests.

In *experiment 2*, to explore the predictive role of baseline oscillations, general linear model analyses were performed separately for online (average changes of each training block compared to the first one) and offline (changes from pre to post) training effects. In addition to condition as within-subject factor, pre-test performance, baseline cpAlpha, fTBR and PAF were included as covariates.

## Results

### Experiment 1: online and offline effects of tDCS on operant tactile learning

With this experiment we aimed at the replication with tDCS of our previous TMS study where we revealed that 15 Hz rTMS over the contralateral SI combined with tactile training facilitates spatial discrimination with the left ring finger (Karim et al. 2006). We expected that anodal tDCS would also have a facilitative effect on tactile training through increasing cortical excitability during training.

### Online effects of tDCS on tactile training

**Fig. 1:**
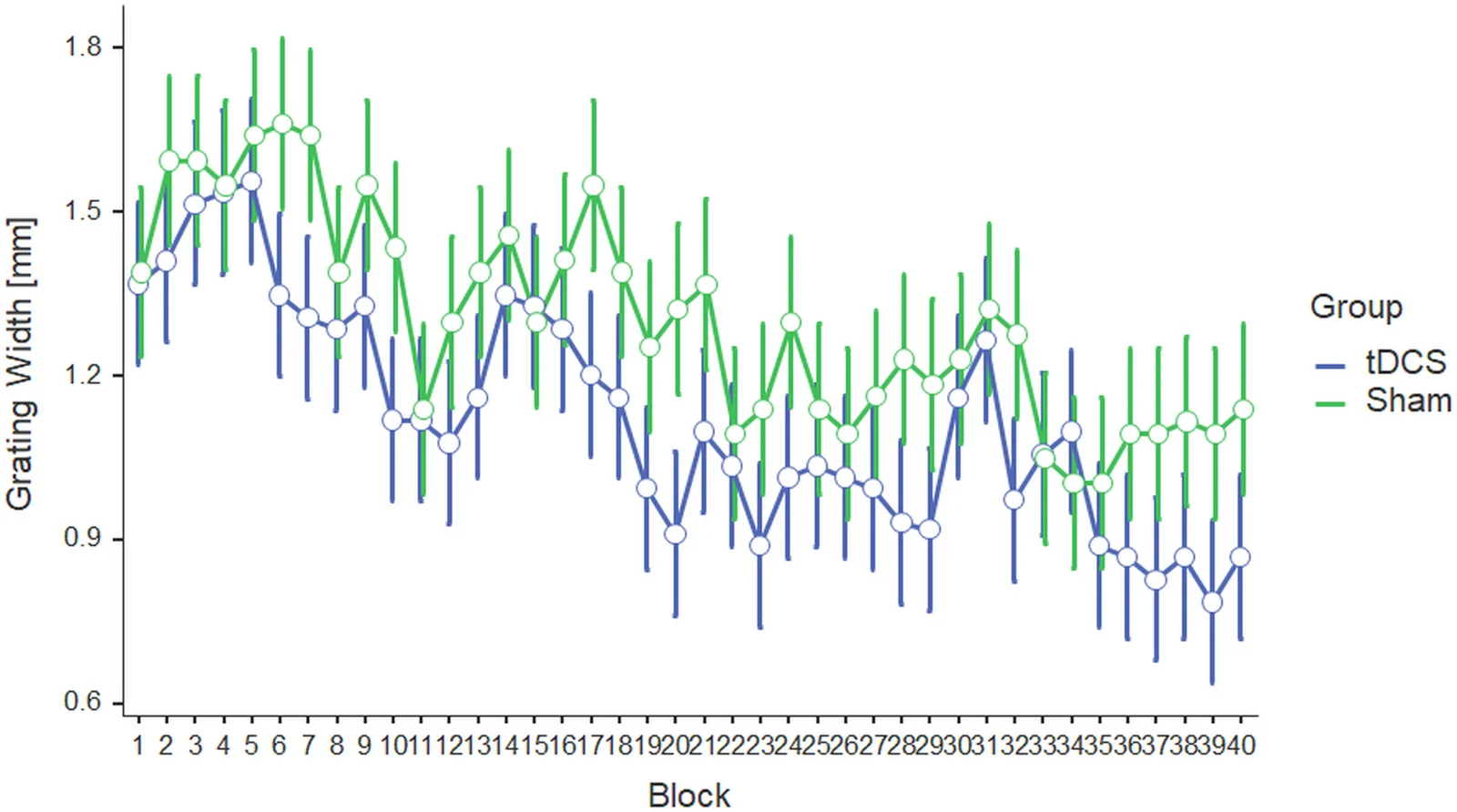
Training effects on GOT grating widths during training in the experimental (tDCS stimulation) and control (sham stimulation) group. Means and standard errors

Both groups, the tDCS and the sham stimulation group, improved during training as indicated by decreasing discrimination widths in the GOT task (Fig. 1). Thresholds for the experimental group were below those of the control group in almost all trials.

Longitudinal analysis of covariance was applied using GLM with grating width used in each block as the outcome variable and GROUP (tDCS versus sham), testing BLOCK and the GROUP x BLOCK interaction as variables in the model adjusted for the PRE-test threshold. The lm() function was used with the following formula:

$$\begin{gathered} {\text{Grating Width}}[{\mathrm{mm}}]\sim {\text{1 + GROUP + PRE + BLOCK + GROUP : BLOCK}} \hfill \\ \end{gathered} $$

The overall model was significant (F(80,839) = 4.21, *p* < .001, adj. R^2^ = 0.218). Significant main effects of GROUP, BLOCK, and PRE but no GROUP x BLOCK interaction effect were revealed, indicating significant training effects for both groups with better overall performance in the tDCS compared to the control group (cf. Table 1 for detailed statistics).

**Table 1:** Comparison of online effects between groups

	SS	df	F	*p*	η^2^*p*
Model	88.19	80	4.21	< 0.001	0.286
Group	6.99	1	26.68	< 0.001	0.031
pre	**50.52**	**1**	**192.76**	**< 0.001**	**0.187**
Block	**32.60**	**39**	**3.19**	**< 0.001**	**0.129**
Group ✻ Block	3.35	39	0.33	1.000	0.015
Residuals	219.88	839			
Total	308.08	919			

Significant effects (p < .05) in bold

Posthoc analysis revealed that the tDCS group performed better in the GOT than the control group (t(839) = 5.16, *p* < .001). Hedge’s g was revealed as 0.398 (95% CI [0.264; 0.53]) indicating a medium sized effect.

**Fig. 2:**
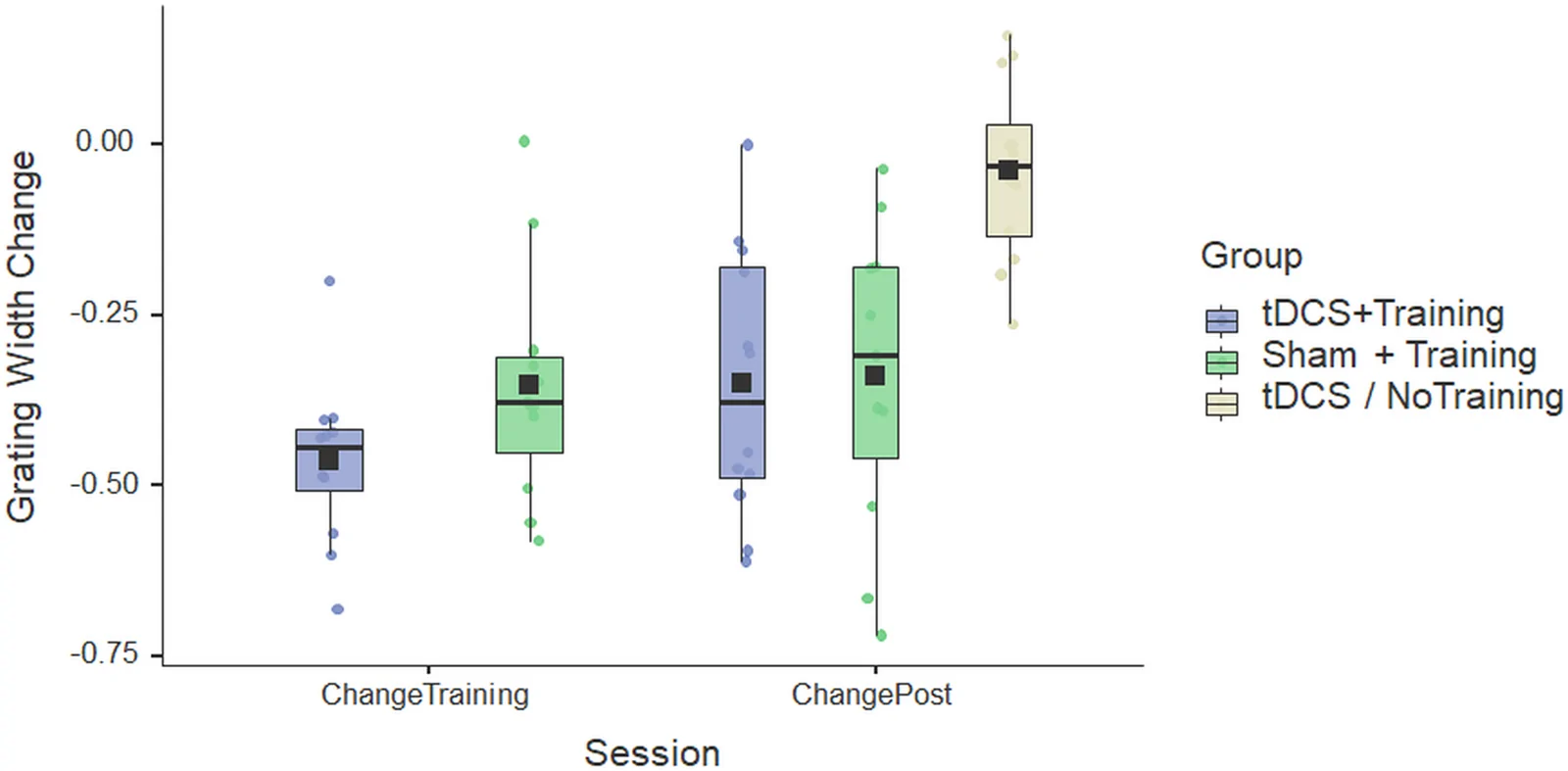
Reduction in grating widths in percent relative to pre-test during training and in the post-test (means, medians, quartiles (boxes) and percentiles (whiskers))

To further quantify the training effects in the tDCS and control groups, we calculated the aggregated change score of the grating width over all 40 blocks as the mean difference per block relative to the pre-test threshold divided by the pre-test threshold. One-way T-tests confirmed that grating widths were significantly reduced by 46% for the tDCS training group (t(11) = 13.34, *p* < .001) and by 35% for the sham training group (t(10) = 6.68, *p* < .001; one-sided one-sample t-tests; cf. Figure 2; Table 2).

**Table 2:** Thresholds and grating widths in the pre-, training, and post-tests

Condition	Pre-test threshold	Training: mean grating width	Post-test threshold
tDCS + Training	2.22 +/− 0.63	1.17 +/− 0.36	1.36 +/− 0.32
Sham + Training	1.99 +/− 0.56	1.25 +/− 0.36	1.35 +/− 0.58
tDCS / No training	1.95 +/− 0.73	–	1.86 +/− 0.73

Given are means and standard deviations in mm

### Offline effects of tDCS and training on tactile perception

From pre- to post-tests, GOT thresholds decreased in both the tDCS and sham stimulation training groups but not in the group that received tDCS but no training (Fig. 2; Table 2).

Longitudinal analysis of covariance was applied using GLM with GOT threshold in mm as the outcome variable and Group (tDCS + Training, Sham + Training, tDCS no training) as variable in the model adjusted for the PRE-test threshold after the following formula:

$$ {\mathrm{Threshold}}[{\mathrm{mm}}] \sim {\text{1 + GROUP + PRE}} $$

The overall model was significant (F(3,31) = 7.41, *p* < .001, adj. R^2^ = 0.361). A significant main effect of GROUP was also revealed, indicating that grating widths were differently affected in the three groups. Posthoc t-tests revealed that both training groups had significantly lower post-test thresholds than the no-training group (Tables 3 and 4). One-way T-tests further confirmed that post-test GOT thresholds were significantly reduced by 35% for the tDCS training group (t(11) = − 6.12, *p* < .001) and by 34% for the sham training group (t(10) = − 5.01, *p* < .001) but not for the no training group (t(11) = − 0.995, *p* = .170; one-sided one-sample t-tests).

**Table 3:** Comparison of offline effects between groups

	SS	df	F	*p*	η^2^*p*
Model	**0.78**	**3**	**7.41**	**< 0.001**	**0.418**
Group	**0.69**	**2**	**9.80**	**< 0.001**	**0.387**
Pre	0.04	1	1.14	0.295	0.035
Residuals	1.09	31			
Total	1.87	34			

Significant effects (p < .05) in bold

**Table 4:** Post Hoc comparisons for offline effects

Comparison	Difference		SE	t	df	p	p_bonferroni_
tDCS+Train. vs. Sham+Training	0.00		0.08	0.02	31	0.983	1.000
tDCS+Train. vs. tDCS/NoTrain.	**0.30**		**0.08**	**3.82**	**31**	**< 0.001**	**0.002**
Sham+Train. vs. tDCS/NoTrain.	**− 0.30**		**0.08**	**3.81**	**31**	**< 0.001**	**0.002**

Significant effects (p < .05) in bold

### Experiment 2: differential effects of tDCS and tACS on tactile operant learning

#### Online effects of tDCS and tACS on tactile training

**Fig. 3:**
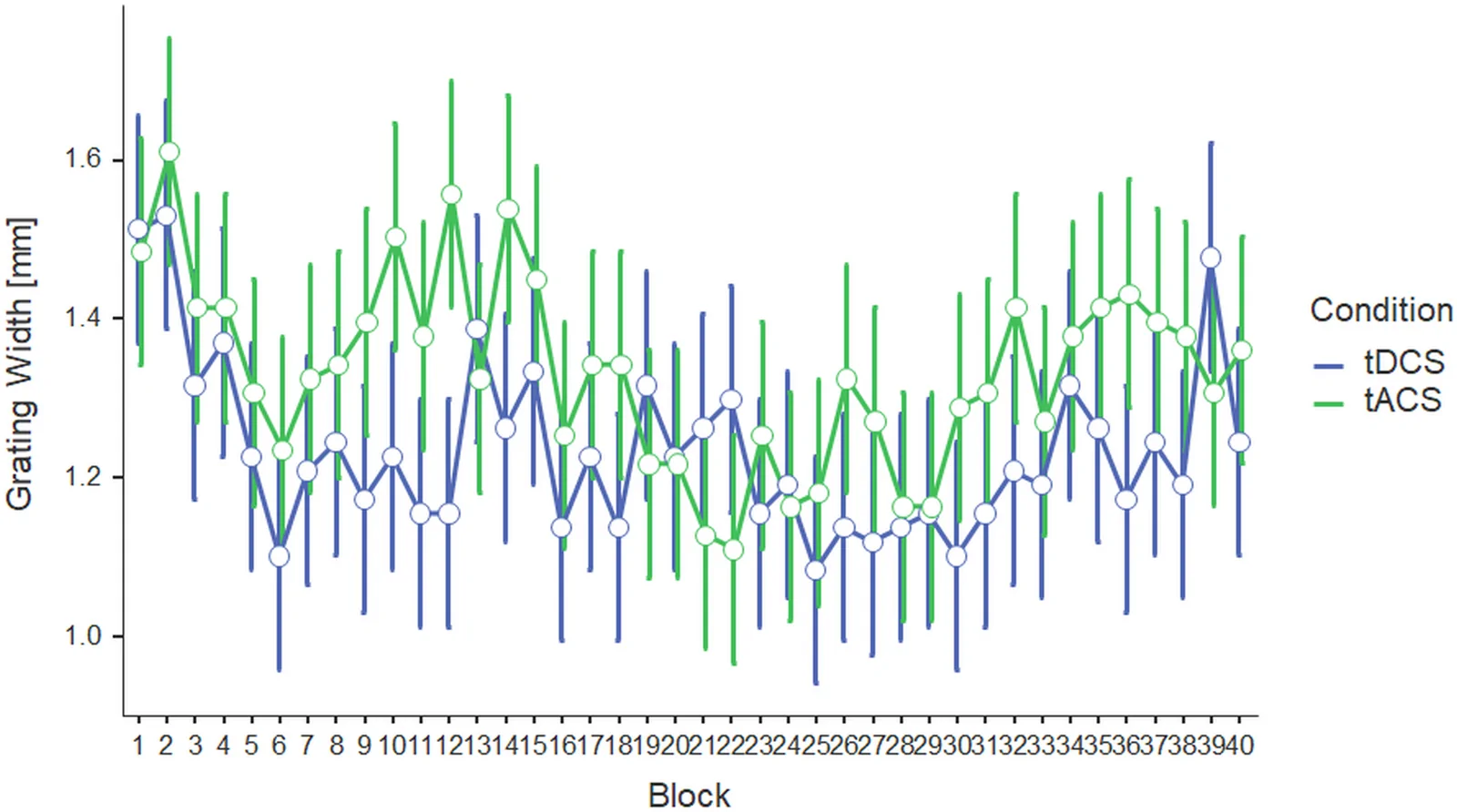
Training effects on GOT thresholds during training in the tDCS and tACS conditions. Means and standard errors

In both conditions, participants improved during training as indicated by decreasing thresholds in the GOT task (Fig. 3). Thresholds for the tDCS conditions were generally below those from the tACS condition.

Longitudinal analysis of covariance was applied using GLM with grating width as the outcome variable and CONDITION (tDCS versus tACS), testing BLOCK and the CONDITION x BLOCK interaction as variables in the model adjusted for the PRE-test threshold using the following formula:

$$\begin{gathered} {\text{Grating Width}}[{\mathrm{mm}}]\sim {\text{1 + CONDITION + PRE + BLOCK + CONDITION}}:{\mathrm{BLOCK}} \hfill \\ \end{gathered} $$

The overall model was significant (F(80,1039) = 3.84, *p* < .001, adj. R^2^ = 0.169). Main effects of CONDITION and PRE-test were revealed, indicating better overall performance in the tDCS compared to the tACS Condition during training (cf. Table 5 for detailed statistics).

**Table 5:** Comparison of online effects between conditions

	SS	df	F	*p*	η^2^*p*
Model	87.59	80	3.84	< 0.001	0.228
Condition	**2.73**	**1**	**9.59**	**0.002**	**0.009**
Block	9.81	39	0.88	0.678	0.032
Pre	**66.10**	**1**	**231.85**	**< 0.001**	**0.182**
Condition ✻ Block	4.25	39	0.38	1.000	0.014
Residuals	296.22	1039			
Total	383.81	1119			

Significant effects (p < .05) in bold

Posthoc analysis revealed that the tDCS group performed better in the GOT than the tACS group (t(1039) = 3.096, *p* = .002). Hedge’s g was revealed as 0.192 (95% CI [0.070; 0.314]) indicating a small to medium sized effect.

To further quantify the training effects, we calculated for both groups the aggregated change scores of the grating width over all 40 blocks as the mean difference per block relative to the pre-test threshold divided by the pre-test threshold.

**Fig. 4:**
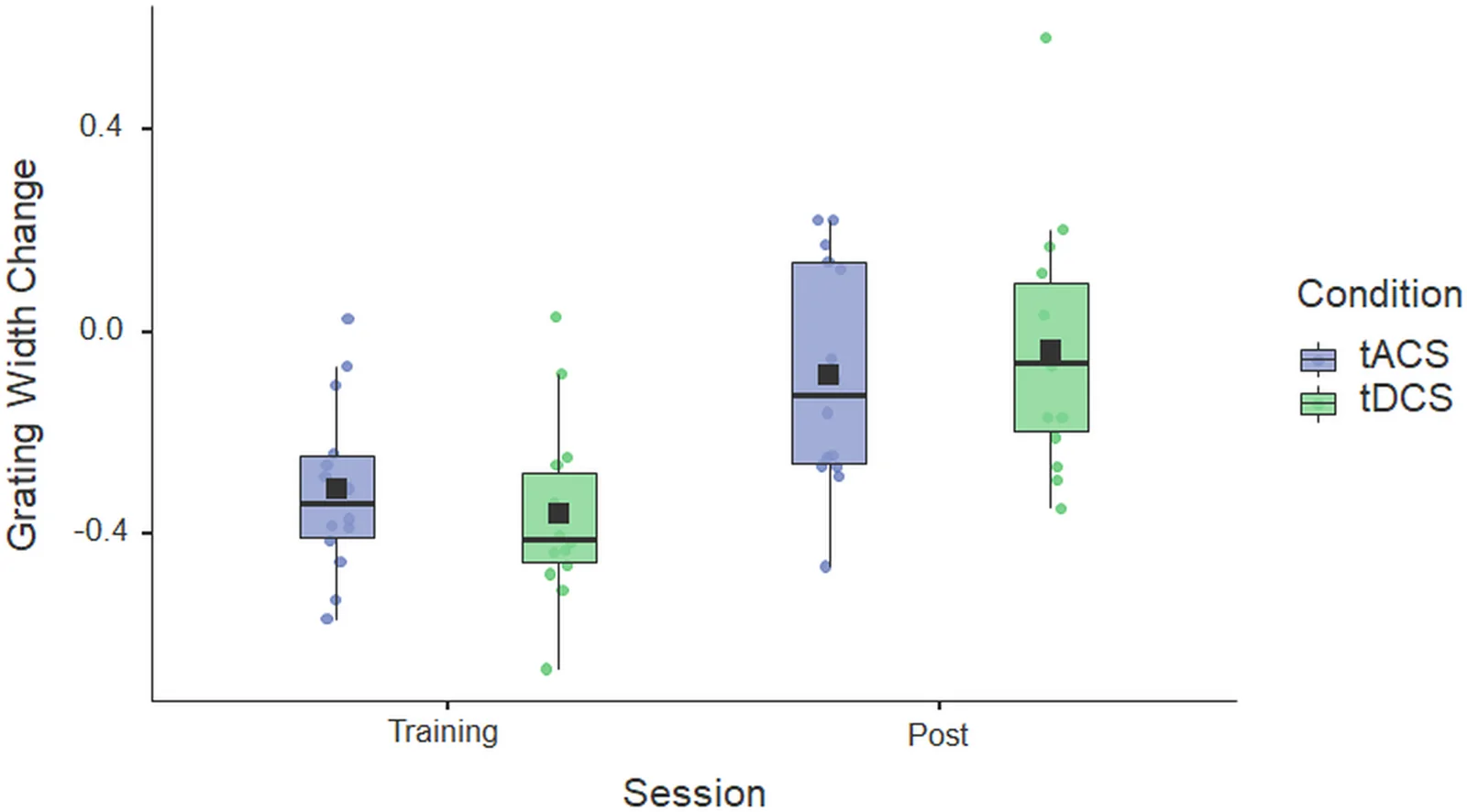
Reduction in grating widths in percent relative to pre-test during training and in the post-test (means, medians, quartiles (boxes) and percentiles (whiskers))

One-way T-tests confirmed that compared to the pre-test grating widths were significantly reduced by 36% for the tDCS condition (t(13) = − 7.64, *p* < .001) and by 31% for the tACS condition (t(13) = − 6.78, *p* < .001; one-sided one-sample t-tests; cf. Figure 4; Table 6).

**Table 6:** Thresholds and grating widths in the pre-, training, and post-tests

Condition	Pre-test threshold	Training mean grating width	Post-test threshold
tDCS	1.91 +/− 0.49	1.20 +/− 0.42	1.80 +/− 0.49
tACS	2.03 +/− 0.47	1.37 +/− 0.39	1.79 +/− 0.38

Given are means and standard deviations in mm

### Offline effects of tDCS and tACS on tactile training

After training, in the post-tests, GOT thresholds returned to baseline values for both stimulation conditions (Fig. 4; Table 7).

Longitudinal analysis of covariance was applied using GLM with post-test GOT thresholds as the outcome variable and CONDITION (tDCS and tACS) as variable in the model adjusted for the PRE-test threshold after the following formula:

$$ {\text{Threshold [mm] }} \sim {\text{ 1 + CONDITION + PRE}} $$

The overall model was significant (F(2,25) = 5.67, *p* = .009, adj. R^2^ = 0.257). Only an effect of PRE-test but no CONDITION effect was revealed (Table 7).

**Table 7:** Comparison of offline effects between conditions

	SS	df	F	*p*	η^2^*p*
Model	1.57	2	5.67	0.009	0.312
Condition	0.04	1	0.27	0.611	0.011
Pre	**1.57**	**1**	**11.33**	**0.002**	**0.312**
Residuals	3.46	25			
Total	5.04	27			

Significant effects (p < .05) in bold

One-way T-tests did not reveal reduced thresholds in the post-test compared to the pre-test (tDCS: t(13) = − 0.548, *p* = .296; tACS: t(13) = − 1.43, *p* = .089; one-sided one-sample t-tests).

### Predictive value of baseline oscillatory EEG activity for online and offline training effects

To examine whether *online* training effects could be predicted by baseline oscillatory brain activity, analysis of covariance was applied using GLM with overall GOT change scores during the training period as dependent variable and CONDITION (tDCS versus tACS) as within-subjects factor in the model adjusted for the PRE-test threshold. cpAlpha, fTBR, and PAF and all interactions with condition were also included in the model resulting in the following formula:

$$\begin{aligned} & \text{Change Score } [\%] \sim 1 + \mathrm{CONDITION} + \mathrm{PRE}\\ & \quad  + \mathrm{cpAlpha} + \mathrm{fTBR} + \mathrm{PAF} \\ & \quad  + (\mathrm{CONDITION} * \mathrm{PRE}) + (\mathrm{CONDITION} * \mathrm{cpAlpha}) \\ & \quad  + (\mathrm{CONDITION} * \mathrm{fTBR}) + (\mathrm{CONDITION} * \mathrm{PAF}) \end{aligned}$$

The overall model was significant (F(9,18) = 2.49, *p* = .0048, adj. R^2^ = 0.331). Main effects of CONDITION and fTBR but no further main or interaction effects were revealed (cf. Table 8, for detailed statistics).

Results confirm stronger online training effects in the tDCS compared to the alpha-aTCS condition. Further, training effects were dependent on frontal TBR with stronger training effects with higher frontal TBR at baseline. As revealed by simple effects analysis, this effect was mainly driven by the tACS condition (tDCS: standardized beta = − 0.334, t = − 1.472, *p* = .158; tACS: standardized beta = − 0.833, t = − 3.364, *p* = .003).

Similarly, to examine whether *offline* training effects could be predicted by baseline oscillatory brain activity, analysis of covariance was applied using GLM with overall change scores in GOT thresholds from pre- to post-test as dependent variable and CONDITION (tDCS versus tACS) as within-subjects factor in the model adjusted for the PRE-test threshold. Again, cpAlpha, fTBR, and PAF and all interactions with condition were also included in the model after the same formula as above.

The overall model was significant (F(9,18) = 2.80, *p* = .030, adj. R^2^ = 0.375; cf. Table 9 for detailed statistics). Results revealed main effects of PRE-test and cpAlpha. Stronger alpha power at baseline predicted stronger offline learning effects in both stimulation conditions. Simple effects analysis revealed that this effect was mainly driven by the tDCS condition (standardized beta = − 0.692, t = − 2.662, *p* = .016) but not the tACS condition (standardized beta = − 0.127, t = − 0.536, *p* = .598). While no further main or interaction effects were revealed, the interaction of CONDITION and fTBR was only marginally significant.

**Table 8:** Online effects dependent on conditions and baseline EEG

	SS	df	F	*p*	η^2^*p*
Model	0.673	9	2.487	0.048	0.554
Pre	0.022	1	0.730	0.404	0.039
**Condition**	**0.232**	**1**	**7.717**	**0.012**	**0.300**
cpAlpha	0.022	1	0.744	0.400	0.040
fTBR	**0.363**	**1**	**12.075**	**0.003**	**0.401**
PAF	0.020	1	0.671	0.423	0.036
Condition ✻ Pre	0.081	1	2.679	0.119	0.130
Condition ✻ cpAlpha	0.000	1	0.014	0.907	0.001
Condition ✻ fTBR	0.066	1	2.210	0.154	0.109
Condition ✻ PAF	0.025	1	0.827	0.375	0.044
Residuals	0.542	18			
Total	1.215	27			

Effect	Estimate	SE	Lower	Upper	β	df	t	*p*
(Intercept)	− 0.291	0.034	− 0.362	− 0.219	− 0.004	18	− 8.509	< 0.001
Pre	0.064	0.075	− 0.093	0.220	0.142	18	0.854	0.404
Condition	**− 0.190**	**0.068**	**− 0.333**	**− 0.046**	**− 0.895**	**18**	**− 2.778**	**0.012**
cpAlpha	− 0.009	0.011	− 0.031	0.013	− 0.157	18	− 0.863	0.400
fTBR	**− 0.089**	**0.026**	**− 0.142**	**− 0.035**	**− 0.583**	**18**	**− 3.475**	**0.003**
PAF	− 0.035	0.043	− 0.125	0.055	− 0.151	18	− 0.819	0.423
Cond.✻ Pre	− 0.244	0.149	− 0.557	0.069	− 0.544	18	− 1.637	0.119
Cond. ✻ cpAlpha	− 0.003	0.021	− 0.047	0.042	− 0.043	18	− 0.119	0.907
Cond.✻ fTBR	0.076	0.051	− 0.031	0.183	0.499	18	1.487	0.154
Cond.✻ PAF	− 0.078	0.085	− 0.257	0.102	− 0.335	18	− 0.909	0.375

Significant effects (p < .05) in bold

**Table 9:** Offline effects dependent on conditions and baseline EEG

	SS	df	F	*p*	η^2^*p*
Model	0.858	9	2.802	0.030	0.584
Pre	**0.462**	**1**	**13.590**	**0.002**	**0.430**
Condition	0.001	1	0.038	0.848	0.002
cpAlpha	**0.185**	**1**	**5.437**	**0.032**	**0.232**
fTBR	0.001	1	0.044	0.837	0.002
PAF	0.040	1	1.168	0.294	0.061
Condition ✻ Pre	0.048	1	1.424	0.248	0.073
Condition ✻ cpAlpha	0.088	1	2.594	0.125	0.126
Condition ✻ fTBR	0.108	1	3.181	0.091	0.150
Condition ✻ PAF	0.001	1	0.024	0.880	0.001
Residuals	0.612	18			
Total	1.470	27			

Effect	Estimate	SE	Lower	Upper	β	df	t	*p*
(Intercept)	− 0.063	0.036	− 0.139	0.013	− 0.009	18	− 1.738	0.099
Condition	0.014	0.073	− 0.138	0.167	0.061	18	0.195	0.848
cpAlpha	**− 0.026**	**0.011**	**− 0.050**	**− 0.003**	**− 0.410**	**18**	**− 2.332**	**0.032**
fTBR	− 0.006	0.027	− 0.063	0.051	− 0.034	18	− 0.209	0.837
PAF	− 0.049	0.045	− 0.144	0.046	− 0.193	18	− 1.081	0.294
Pre	**− 0.292**	**0.079**	**− 0.458**	**− 0.126**	**− 0.592**	**18**	**− 3.686**	**0.002**
Cond. ✻ fTBR	− 0.097	0.054	− 0.211	0.017	− 0.579	18	− 1.784	0.091
Cond.✻ cpAlpha	− 0.036	0.023	− 0.084	0.011	− 0.566	18	− 1.611	0.125
Cond.✻ PAF	0.014	0.091	− 0.177	0.205	0.055	18	0.154	0.880
Cond.✻ Pre	0.189	0.158	− 0.144	0.522	0.383	18	1.193	0.248

Significant effects (p < .05) in bold

### Comparison of tDCS and TMS effects on tactile training

To compare online and offline effects of brain stimulation in this study with previously published TMS effects (Karim et al. 2006), we recalculated Hedge’s g from the original TMS data.

The effect sizes for the comparison between real and sham stimulation on grating widths during training as revealed from experiment 1 and the previously reported TMS study were comparable and indicated medium sized effects for both studies (Table 10). The effect size in experiment 2, comparing tDCS to tACS, was a bit smaller but still significant and 95% confidence intervals overlapped with the effects of the other two experiments (Table 10). As described above, no offline training effects were found in experiment 1 and 2 and there was no significant difference between the tDCS and tACS conditions in the post-test. However, the comparison of TMS and sham stimulation effects on threshold changes in the post-test revealed a strong and significant offline effect of TMS stimulation combined with training.

**Table 10:** Comparison of tDCS/tACS and TMS effects

Training				95% Confidence intervals	
Comparison	Difference	SE	g_sample_	Lower	Upper
[1] tDCS - Sham	− 0.21	0.04	− 0.40	− 0.53	− 0.26
[2] tDCS - tACS	− 0.10	0.03	− 0.19	− 0.31	− 0.07
[3] TMS - Sham	− 0.18	0.04	− 0.32	− 0.47	− 0.17

Post-test				95% Confidence intervals	
Comparison	Difference	SE	g_sample_	Lower	Upper
[1] tDCS - Sham	− 0.00	0.08	− 0.01	− 0.69	0.68
[2] tDCS - tACS	− 0.02	0.08	− 0.08	− 0.84	0.68
[3] TMS - Sham	− 0.22	0.10	− 0.88	− 0.07	− 1.67

[1]: Experiment 1, [2]: Experiment 2, [3]: Karim et al. 2006

## Discussion

In separate experiments we examined the effects of transcranial electrical stimulation on tactile operant learning in a grating orientation discrimination task. Our aim was to (1) investigate whether the previously reported facilitative effects of TMS could be replicated using tDCS, while also comparing their respective effect sizes, (2) to examine whether tACS is as effective or more effective than tDCS, and (3) whether effects of tDCS and tACS on tactile learning can be predicted by baseline EEG oscillatory power. While in experiment 1 we compared the online and offline training effects of tDCS to sham stimulation and tDCS effects without training, in experiment 2, we contrasted the effects of tDCS and tACS on tactile training and examined whether training effects could be predicted by baseline oscillatory brain activity.

### Is tDCS as effective as TMS in facilitating operant tactile learning in the GOT task?

In both experiments, participants improved their discrimination performance during training independent of group (experiment 1) or condition (experiment 2), even when controlling for pre-test thresholds. As predicted, the tDCS training group revealed significantly lower GOT thresholds than the sham training group in experiment 1. Effect size was in a similar range as in our previous TMS study (Karim et al. 2006), confirming that tDCS and TMS over the contralateral somatosensory cortex have comparable online effects on tactile learning despite different neurophysiological mechanisms underlying those effects.

Offline effects were inconsistent between experiments, and online training effects did not translate directly into offline effects. While in experiment 1 training effects persisted partly in the post training test for both training groups (tDCS and sham), in experiment 2, neither the tDCS nor the tACS condition revealed any offline effect of the stimulation in the post-test. While the training protocol and the tDCS stimulation paradigm was identical between experiments, the only differences were the concurrent EEG measurements in experiment 2 which also required a different placement of the refererence electrode. With respect to the electrode setup one might argue that with a frontal reference the electrical field over the somatosensory cortex extends more into the frontal lobe while with a reference over the neck it would extend more in the direction of the occipital lobe. However, according to Nitsche and colleagues current density determines the efficacy of tDCS and by using a small stimulation electrode and a large reference electrode as in our experiments modulation of cortical excitability can be focused to the target area (Nitsche and Paulus 2000; Nitsche et al. 2007). We therefore suggest that the EEG setup in experiment 2 imposes mental stress and participants getting tired earlier than in experiment 1. This argument is supported by the decreased performance in the later training trials for both conditions and might also connect to differences in the offline effect.

Moreover, even in experiment 1 the combination of training with tDCS did not result in a stronger offline training effect compared to training with sham stimulation. These results contrast with the results from our previous TMS study in which marked offline effects of TMS compared to sham stimulation were revealed (Karim et al. 2006). One might argue that online modulation of neuronal cell membranes through transcranial electrical stimulation has a facilitative effect on somatosensory processing – tDCS more than tACS – resulting in reduced discrimination thresholds during the learning period. Different to TMS, however, aftereffects of the stimulation seem to be weak or even absent.

#### Is tACS as effective as tDCS in facilitating operant tactile learning in the GOT task?

Saito et al. (2021), Feurra et al. (2011) and Millard et al. (2024) reported that alpha oscillations relate to enhanced gating of sensory information and facilitate sensory training effects. Thus, we assumed that alpha-tACS enhancing alpha oscillations could also have positive effects on tactile learning. However, in experiment 2, participants revealed lower GOT thresholds during training in the tDCS than in the tACS condition. This result seems to be in line with studies reporting reduced tactile detection after alpha-tACS (Sliva et al. 2018) or revealing that pre-stimulus alpha was associated with impaired stimulus detection after cued attention (Jones et al. 2010). One might speculate that tACS interferes with attention-induced task-related alpha desynchronization during training. Saito et al. (2021) also found that participants with low alpha at baseline profited most from the tACS, indicating that individual differences might explain some inconsistencies and that there might be an optimal individual alpha level. In addition, Gundlach et al. (2016) argued that the effect of tACS on sensory gating is phase dependent. Further investigations are required combining alpha-tACS with EEG during training to solve the described discrepancies on a mechanistic level. It is important to note that our design does not allow any conclusion about potential effects of tACS on tactile discrimination performance compared to a no-stimulation control. The observed difference between tDCS and tACS during training could reflect several scenarios: tDCS facilitating performance while tACS has no effect, tACS interfering with performance while tDCS has no effect, or both stimulation types having opposing effects relative to no stimulation. It cannot be determined from the current design which of these accounts is correct.

#### Can online and/ or offline training effects be predicted by baseline EEG?

Irrespective of stimulation conditions (tDCS, tACS, sham), training effects could be predicted by baseline brain oscillatory patterns. Higher frontal TBR but not centroparietal alpha power at baseline predicted stronger online training effects. This fits well with a recent study on dynamic balance training (Imani and Godde 2025). After Begus and Bonawitz (2020) and Nokia et al. (2012), baseline theta oscillations can serve as a useful neural marker to predict the capacity for new learning.

Outside the tactile domain, resting-state beta oscillations (13–14.5 Hz) were shown to predict language proficiency and learning success in young (Kliesch et al. 2022) and older adults learning a second language (Kliesch et al. 2021). Another study found that lower levels of resting-state theta power (6–7 Hz) were associated with better implicit visuospatial-motor sequence learning (Lum et al. 2023). In cognitive training, a higher alpha/theta ratio was associated with better outcomes (Juras et al. 2025).

On the individual level, consistent with the literature (Muller-Gass et al. 2017; Brickwedde et al. 2019), higher centroparietal alpha power during baseline also predicted stronger offline training effects, particularly in the tDCS condition. We expected that entrainment of alpha oscillations through 10 Hz-tACS might have similar gating effects on learning as upregulating alpha power using neurofeedback (Brickwedde et al. 2019). This view fits with a report by Saito et al. (2019) revealing that alpha-frequency tACS effects on improving tactile performance were greater in those participants having lower baseline alpha power. However, resting state alpha power predicted learning predominantly in the tDCS condition and the individual peak alpha frequency did not modulate the training effect in the tACS condition. Thus, specific entrainment to 10 Hz alpha oscillations through tACS seems unlikely as facilitator of tactile learning in the current experiments.

A higher frontal theta-beta ratio and reduced low-beta power predicted stronger offline training effects specifically in the tDCS condition, although this did not significantly exceed the tACS condition. Given that alpha-frequency tACS may cause temporary perceptual suppression (Sliva et al. 2018), 10-Hz tACS might have neutralized the facilitative effects of higher theta-beta ratio. Nevertheless, because these findings were only marginally significant, they must be interpreted cautiously.

#### Limitations

A major limitation of this study is the small sample size. This limits particularly the validity of individual contributions for conclusions on the general population and also affects effect size estimations (Minarik et al. 2016). The individual differences analyses in Experiment 2 are particularly affected by the small sample size. These findings should be regarded as exploratory and hypothesis-generating, pending replication in larger samples.

Another limitation refers to missing control groups or conditions. In experiment 1 as control group we had an active learning group receiving sham stimulation but no active learning group without stimulation. Given that sham stimulation should not have any effect, we however argue that it is equivalent to a no-stimulation active control group. In experiment 2 we focused on the comparison between tDCS and tACS. The chosen design does not allow any conclusion about potential effects of tACS on tactile discrimination performance compared to a no-stimulation control and the finding that neither the tDCS nor the tACS condition revealed any offline effect in the post-test must not be interpreted as that there are no effects. The absence of a sham condition applies equally to online training effects. The superiority of tDCS over tACS during training cannot be interpreted as evidence that tDCS facilitated learning, since tACS may have actively interfered with performance rather than being inert.

A further limitation concerns blinding, particularly in Experiment 2. Because participants completed both tDCS and tACS sessions, they may have been able to distinguish between conditions based on differences in subjective sensation. tACS at alpha frequency can produce a distinct flickering or tingling quality that differs from the more constant sensation of tDCS. No post-session blinding check was conducted to verify whether participants correctly identified their condition, so the integrity of blinding in Experiment 2 cannot be confirmed. Future studies comparing tDCS and tACS should include a standardized blinding assessment after each session.

### Concluding remarks

Combined, the inconsistencies reported in the literature about effects of tDCS and tACS on tactile training (cf. Khalil et al. 2023) are also present among the two experiments of the current study. One might argue that facilitative effects of transcranial electrical stimulation and particularly interaction effects of stimulation condition with resting EEG are too small to be consistently revealed with the sample size of this study and of mostly all other studies done so far. Much larger samples are particularly needed because not only training effects in general but also the additional and facilitative effect of brain stimulation depends on individual characteristics, such as individual learning thresholds (cf., Dinse et al. 2003) and baseline oscillatory brain activity patterns interacting with the applied modulation through tDCS or tACS. Further research is necessary to identify which participants would profit most from the combination of training with brain stimulation.

## Data Availability

All research data will be made publicly available after publication.
